# Clinical Characteristics of Patients With Progressive and Non-progressive Coronavirus Disease 2019: Evidence From 365 Hospitalised Patients in Honghu and Nanchang, China

**DOI:** 10.3389/fmed.2020.556818

**Published:** 2020-11-16

**Authors:** Yanpei Zhang, Lu-shan Xiao, Pu Li, Hongbo Zhu, Chenyi Hu, Wen-Feng Zhang, Qing-can Sun, Meng-ying Shen, Shan-shan Liu, Wan-li Zhang, Han-yi Zeng, Mengchun Gong, Li Liu, Yu-Lin He, Hong Zhu

**Affiliations:** ^1^Department of Medical Quality Management, Nanfang Hospital, Southern Medical University, Guangzhou, China; ^2^Department of Infectious Diseases, Nanfang Hospital, Southern Medical University, Guangzhou, China; ^3^State Drug Clinical Trial Agency, The First Affiliated Hospital, Nanchang University, Nanchang, China; ^4^Department of Oncology, The First Affiliated Hospital of University of South China, Hengyang, China; ^5^Department of Infectious Diseases, The First Affiliated Hospital, Nanchang University, Nanchang, China; ^6^Digital China Health Technologies Corporation Limited, Beijing, China; ^7^Department of Radiology, The First Affiliated Hospital, Nanchang University, Nanchang, China; ^8^Health Management Institute, Nanfang Hospital, Southern Medical University, Guangzhou, China

**Keywords:** coronavirus disease 2019, disease progression, severe acute respiratory syndrome coronavirus 2, bilateral patchy shadowing, creatinine kinase, creatinine, C-reactive protein

## Abstract

**Background:** Coronavirus disease (COVID-19) has swept around the globe and led to a worldwide catastrophe. Studies examining the disease progression of patients with non-severe disease on admission are scarce but of profound importance in the early identification of patients at a high risk of deterioration.

**Objectives:** To elucidate the differences in clinical characteristics between patients with progressive and non-progressive COVID-19 and to determine the risk factors for disease progression.

**Study design:** Clinical data of 365 patients with non-severe COVID-19 from 1 January 2020 to 18 March 2020 were retrospectively collected. Patients were stratified into progressive and non-progressive disease groups. Univariate and multivariate logistic regression analyses were performed to determine the independent risk factors for disease progression.

**Results:** Compared with patients with non-progressive disease, those who progressed to severe COVID-19 were older and had significantly decreased lymphocyte and eosinophil counts; increased neutrophil and platelet counts; lower albumin levels; higher levels of lactate dehydrogenase, C-reactive protein (CRP), creatinine, creatinine kinase, and urea nitrogen; and longer prothrombin times. Hypertension, fever, fatigue, anorexia, bacterial coinfection, bilateral patchy shadowing, antibiotic and corticosteroid administration, and oxygen support had a significantly higher incidence among patients with progressive disease. A significantly longer duration of hospital stay was also observed in patients with progressive disease. Bilateral patchy shadowing (OR = 4.82, 95% CI: 1.33–17.50; *P* = 0.017) and elevated levels of creatinine (OR =6.24, 95% CI: 1.42–27.40; *P* = 0.015), and CRP (OR = 7.28, 95% CI: 2.56–20.74; *P* < 0.001) were independent predictors for disease progression.

**Conclusion:** The clinical characteristics of patients with progressive and non-progressive COVID-19 were significantly different. Bilateral patchy shadowing and increased levels of creatinine, and CRP were independent predictors of disease progression.

## Introduction

Coronavirus disease (COVID-19) has swept around the world, with more than 2.9 million confirmed cases resulting in 204,000 deaths as of 27 April 2020. Its pathogen, severe acute respiratory syndrome coronavirus 2 (SARS-CoV-2), can spread rapidly between humans, causing the number of confirmed cases to increase quickly. The severity spectrum of COVID-19 ranges from mild to critical, but the majority of infections have not been severe (1–6). Eighty-one percent of confirmed cases have been mild to moderate, including those with and without pneumonia; 14% had severe disease (dyspnoea, blood oxygen saturation ≤ 93% at rest, PaO_2_/FiO_2_ ratio ≤ 300 mmHg, respiratory rate ≥ 30 breaths/min, and lung infiltrates appearing within 24–48 h in > 50% of the lung field); and 5% have presented in critical condition (septic shock, respiratory failure, and/or multiple organ dysfunction) (7). It has been reported that mild symptoms may become severe within 5–7 days (1, 7), suggesting that patients initially hospitalised with mild or moderate disease are still at a risk of severe or critical illness. If the treatment of severely ill patients lies “downstream” to the control of COVID-19, then it is the “upstream” strategy to identify patients who are at a risk of progressing to severe disease. Early identification of these patients coupled with early intervention could save lives and alleviate the burden on the health care system.

Previous studies placed huge significance on transmission dynamics (8, 9), the clinical manifestations of COVID-19 (3, 4, 7), characteristics of patients with severe/critical disease (6, 10), and the differences between patients with severe and non-severe disease (11, 12). However, studies shedding light on patients with non-severe disease on admission and tracking their disease progression are scarce but of profound importance. Therefore, in this study, we tracked patients admitted with mild or moderate COVID-19 and probed the discrepancies between progressor and non-progressor patients to determine the risk factors for disease progression. In this way, we hope to guide the development of effective early predictive tools to identify patients at a high risk for developing severe disease.

## Materials and Methods

### Study Design and Patient Cohort

This retrospective study was approved by the Medical Ethics Committee of Nanfang Hospital of Southern Medical University. Written informed consent from all participating patients was obtained.

We retrospectively collected data from 690 patients with confirmed COVID-19 diagnosed between 1 January 2020 and 18 March 2020 from hospitals in Honghu and Nanchang. Patients were diagnosed according to the guidelines of the Diagnosis and Treatment Protocol for Novel Coronavirus Pneumonia (Trial Version 7) (13). SARS-CoV-2 nucleic acid tests were positive in all participants, and all diagnostic criteria were met according to the guidelines. After excluding patients who had incomplete clinical data, 442 patients were retained, among whom 365 patients with mild or moderate disease were included in the final analysis. Of the 365 patients, 285 were from the People's Hospital of Honghu, while 80 were from the First Affiliated Hospital of Nanchang University.

### Definition

All patients were diagnosed and typed under the guidance of the Diagnosis and Treatment Protocol for Novel Coronavirus Pneumonia (Trial Version 7) (13) developed by the Chinese National Health Commission and the State Administration of Traditional Chinese Medicine. Patients with mild symptoms and no signs of pneumonia on imaging were regarded to have mild disease, while fever and respiratory symptoms were indicators of moderate disease. Patients who suffered from respiratory distress, arterial partial pressure of oxygen (PaO_2_)/fraction of inspired oxygen (FiO_2_) ≤ 300 mmHg, oxygen saturation ≤ 93% at rest, or with obvious lesion progression on chest imaging within 24–48 h of >50%, were considered to have severe disease. When respiratory failure, shock, or other organ failure appeared, patients were diagnosed with critical disease. In this study, patients who maintained mild or moderate symptoms during the entire hospital stay were assigned to the non-progressive disease group, and those with mild or moderate disease on admission who later progressed to a severe or critical status were assigned to the progressive disease group.

### Data Collection and Outcome Evaluation

Clinical electronic medical records, nursing records, laboratory findings, and radiological reports for all included patients with COVID-19 were reviewed. For each patient, detailed admission data were collected, including demographic information, signs and symptoms, comorbidities, imaging reports, and laboratory test results. After admission, the treatment, disease severity state, outcomes, and length of hospital stay were also recorded. The treatments were conducted before disease progression. Two researchers independently checked the electronic medical reports and recorded the daily assessment of the disease severity. The progression from non-severe to severe disease was monitored.

### Statistical Analysis

Continuous variables are presented as mean and standard deviation (SD) while normally distributed and otherwise as medians and interquartile ranges (IQR). Categorical variables are presented as frequencies and percentages. For continuous variables, the comparisons between progressive and non-progressive disease groups were performed using the Student's *t*-test or the Mann–Whitney U test, as appropriate. For categorical variables, comparisons were conducted using the Pearson's chi-square test or Fisher's exact test, as appropriate. To investigate the risk factors associated with disease progression, univariate and multivariate logistic regression models were used. In the univariate logistic regression analyses, variables with *P* < 0.05 were regarded as potential risk factors and included in multivariate regression analysis by a backward elimination procedure (likelihood ratio test and elimination if *P* > 0.1). Statistical analyses were conducted with the SPSS software version 25.0 and R version 4.0.2. The pROC package was employed to draw the receiver operating characteristic (ROC) curves and calculate the area under the curve (AUC). All statistical tests were two-sided, and *P* < 0.05 was regarded as statistically significant.

## Results

By 18 March 2020, a total of 690 patients with confirmed COVID-19 were recruited, of whom 365 patients from the People's Hospital of Honghu and the First Affiliated Hospital of Nanchang University with complete medical records and who were diagnosed with mild or moderate COVID-19 on admission were included in this study.

The demographics and baseline clinical characteristics of these patients are shown in Tables 1, 2. The patients' mean age was 46.8 years [SD 15.5], 74 patients (20.3%) were older than 60 years, and 176 (48.2%) were men. Only 7 (1.9%) were current smokers. Hypertension (10.7%) was the most commonly observed comorbidity, followed by diabetes mellitus (4.7%), chronic liver disease (2.5%), and cardiovascular disease (1.6%). Among these patients, 221 (60.5%) had a fever, 194 (53.2%) had a cough, 253 (69.3%) had multi-lobular infiltration, and 228 (62.5%) had bacterial co-infection. The patients' median temperature on admission was 36.8 degrees Centigrade (IQR 36.5–37.1). As for treatment, 352 (96.4%) received antiviral therapy, 251 (68.8%) underwent antibiotic therapy, 131 (35.9%) were treated with corticosteroids, and 149 (40.8%) required oxygen support. In terms of the outcomes, 363 patients (99.5%) were ultimately discharged from the hospital, and 2 patients (0.5%) died during their hospital stay. The median length of hospital stay was 14 days (IQR 10–20).

**Table 1:** Demographics and baseline characteristics of patients with non–severe COVID-19.

**Characteristic**	**All patients (*n* = 365)**
Age, years	46.8 ± 15.5
≥60	74 (20.3)
Male	176 (48.2)
**Laboratory findings**	
Red blood cell count, × 10^9^/L	4.5 (4.1–4.9)
White blood cell count, × 10^9^/L	6.0 (4.3–6.9)
<10	346 (94.8)
Lymphocyte count, × 10^9^/ L	1.5 (1.1–1.8)
<0.8	36 (9.9)
Neutrophil count, × 10^9^/L	4.0 (2.5–4.7)
>6.4	32 (8.8)
Eosinophil counts, × 10^9^/L	0.1 (0.0–0.1)
Hemoglobin, g/dL	135.3 (125.0–147.0)
Platelet count, × 10^9^/L	241.4 (178.0–293.5)
<100	9 (2.5)
Albumin, g/L	42.7 (38.1–44.9)
<34	27 (7.4)
Total bilirubin, μmol/L	11.1 (7.3–13.4)
>17.1	44 (12.1)
Direct bilirubin, μmol/L	3.4 (2.2–3.8)
Alanine aminotransferase, U/L	30.6 (13.0–34.5)
>40	66 (18.1)
Prothrombin time, s	12.6 (11.9–13.2)
≥16	4 (1.1)
Creatinine, μmol/L	65.6 (50.9–74.2)
≥133	10 (2.7)
Urea nitrogen, mmol/L	4.6 (3.3–5.1)
Lactate dehydrogenase, U/L	219.4 (167.5–246.0)
>245	92 (25.2)
Creatinine kinase, U/L	94.0 (44.5–103.0)
>200	27 (7.4)
C-reactive protein, mg/L	16.3 (0.5–15.2)
≥10	109 (29.9)
**Abnormalities on chest CT**	
Ground-glass opacity	181 (49.6)
Local patchy shadowing	55 (15.1)
Bilateral patchy shadowing	200 (54.8)
Interstitial abnormalities	11 (3.0)

*Continuous variable data are presented as the mean (standard deviation) or median (interquartile ranges, IQR). Classified variable data are presented as n (%)*.

**Table 2:** Smoking history, comorbidity, signs and symptoms, treatment and clinical outcomes of patients with non-severe COVID-19.

**Characteristic**	**All patients (*n* = 365)**
**Smoking history**	
Current smokers	7 (1.9)
Ex-smokers	0 (0.0)
**Comorbidity**	
Hypertension	39 (10.7)
Diabetes mellitus	17 (4.7)
Cardiovascular disease	6 (1.6)
Cerebrovascular disease	3 (0.8)
Chronic liver disease	9 (2.5)
COPD	2 (0.5)
Asthma	1 (0.3)
Renal disease	2 (0.5)
Cancer	2 (0.5)
**Signs and symptoms**	
Fever	221 (60.5)
Temperature on admission (°C)	36.8 (36.5–37.1)
Highest temperature (°C)	37.4 (36.6–38.0)
<37.3	192 (52.6)
37.3–37.9	61 (16.7)
38–38.9	92 (25.2)
≥39	20 (5.5)
Cough	194 (53.2)
Sputum production	52 (14.2)
Nasal congestion	6 (1.6)
Fatigue	77 (21.1)
Headache	17 (4.7)
Sore throat	37 (10.1)
Shortness of breath	28 (7.7)
Dyspnea	13 (3.6)
Anorexia	27 (7.4)
Diarrhea	15 (4.1)
Nausea	13 (3.6)
Myalgia or arthralgia	1 (0.3)
Combination of bacterial infection	228 (62.5)
**Treatment**	
Antiviral therapy	352 (96.4)
Antibiotic therapy	251 (68.8)
Use of corticosteroid	131 (35.9)
Oxygen support	149 (40.8)
**Clinical outcomes**	
Discharge from hospital	363 (99.5)
Length of hospital stay	14.0 (10.0–20.0)
Death	2 (0.5)

*Continuous variable data are presented as the mean (standard deviation) or median (interquartile ranges, IQR). Classified variable data are presented as n (%). COPD, Chronic obstructive pulmonary disease*.

The patients were divided into progressive and non-progressive disease groups, depending on whether their disease became severe after admission. The results of the comparisons between groups are shown in Tables 3, 4. Compared with patients with non-progressive disease, those who progressed to severe COVID-19 were older (59.3 years [SD 13.2] vs. 45.9 years [SD 15.3], *P* < 0.001). The proportion of patients with hypertension, fever, fatigue, anorexia, bacterial co-infection, or bilateral patchy shadowing was significantly higher among patients with progressive disease. From the perspective of laboratory findings, we found significantly decreased lymphocyte and eosinophil counts; increased neutrophil counts; lower albumin levels; higher levels of lactate dehydrogenase, C-reactive protein (CRP), creatinine, creatinine kinase, and urea nitrogen; and a longer prothrombin time in patients with progressive disease than in patients with non-progressive disease. Notably, a significantly higher ratio of patients with progressive disease received antibiotic therapy and had corticosteroids and oxygen support administered. A significantly longer length of hospital stay was also observed in patients with progressive disease.

**Table 3:** Demographics and clinical characteristics of progressive and non-progressive COVID-19 patients.

	**Disease progression**		
**Characteristic**	**Non-progressor patients (*n* = 339)**	**Progressor patients (*n* = 26)**	***P*-value**
Age, years	45.9 ± 15.3	59.3 ± 13.2	<0.001
≥60	63 (18.6)	11 (42.3)	0.004
Male	157 (46.3)	19 (73.1)	0.008
**Laboratory findings**			
Red blood cell count, × 10^9^/L	4.5 (4.1–4.8)	4.5 (3.9–5.0)	0.857
White blood cell count, × 10^9^/L	5.5 (4.3–6.8)	6.1 (4.3–7.6)	0.352
<10	323 (95.3)	23 (88.5)	*p* = 0.131
Lymphocyte count, × 10^9^/ L	1.47 (1.1–1.6)	0.9 (0.7–1.5)	<0.001
<0.8	29 (8.6)	7 (26.9)	0.001
Neutrophil count, × 10^9^/L	3.4 (2.4–4.6)	4.0 (2.7–6.5)	0.033
>6.4	25 (7.4)	7 (26.9)	0.001
Eosinophil counts, × 10^9^/L	0.05 (0–0.1)	0.01 (0–0.1)	0.002
Hemoglobin, g/dL	135.0 (125.0–147.0)	141.0 (120.8–154.5)	0.445
Platelet count, × 10^9^/L	236.0 (180.0–297.8)	197.0 (141.8–233.5)	0.004
<100	7 (2.1)	2 (7.7)	0.075
Albumin, g/L	42.0 (38.5–45.0)	38.2 (34.3–42.8)	0.004
Total bilirubin, μmol/L	9.8 (7.3–13.3)	11.3 (7.2–15.4)	0.315
>17.1	38 (11.2)	6 (23.1)	0.108
Alanine aminotransferase, U/L	20.0 (13.0–35.0)	29.0 (17.0–35.0)	0.085
>40	63 (18.6)	3 (11.5)	0.596
Prothrombin time, s	12.5 (11.8–13.1)	13.0 (12.4–13.8)	0.003
≥16	3 (0.9)	1 (3.8)	0.257
Creatinine >133μmol/L	6 (1.8)	4 (15.4)	<0.001
Lactate dehydrogenase, U/L	199.0 (167.0–241.0)	248.5 (208.0–342.8)	<0.001
>245	78 (23.0)	14 (53.8)	<0.001
Creatinine kinase, U/L	67.0 (44.0–95.0)	108.0 (60.3–192.3)	0.011
>120	56 (16.5)	11 (42.3)	0.003
C-reactive protein, mg/L	2.0 (0.5–12.3)	37.7 (9.6–59.9)	<0.001
≥10	89 (26.3)	20 (76.9)	
**Abnormalities on chest CT**			
Ground-glass opacity	166 (49.0)	15 (57.7)	0.391
Local patchy shadowing	53 (15.6)	2 (7.7)	0.397
Bilateral patchy shadowing	177 (52.2)	23 (88.5)	<0.001
Interstitial abnormalities	11 (3.2)	0 (0)	>0.999

*Continuous variable data are presented as the mean (standard deviation) or median (interquartile ranges, IQR). Classified variable data are presented as n (%)*.

**Table 4:** Smoking history, comorbidity, signs and symptoms, treatment and clinical outcomes of progressive and non-progressive COVID-19 patients.

	**Disease progression**		
**Characteristic**	**Non-progressor patients (*n* = 339)**	**Progressor patients (*n* = 26)**	***P*-value**
**Smoking history**			
Current smokers	5 (1.5)	2 (7.7)	0.082
Ex-smokers	0 (0)	0 (0)	
**Comorbidity**			
Hypertension	33 (9.7)	6 (23.1)	0.046
Cardiovascular disease	4 (1.2)	2 (7.7)	0.061
Cerebrovascular disease	2 (0.6)	1 (3.8)	0.199
Chronic liver disease	8 (2.4)	1 (3.8)	0.49
COPD	2 (0.6)	0 (0)	>0.999
Asthma	1 (0.3)	0 (0)	>0.999
Renal disease	2 (0.6)	0 (0)	>0.999
Cancer	2 (0.6)	0 (0)	>0.999
**Signs and symptoms**			
Fever	198 (58.4)	23 (88.5)	0.003
Temperature on admission (°C)	36.7 (36.5–37.1)	37.1 (36.7–38.0)	0.001
Highest temperature (°C)	37.1 (36.6–38.0)	38.3 (37.7–38.6)	<0.001
<37.3	188 (55.5)	4 (15.4)	<0.001
37.3–37.9	57 (16.8)	4 (15.4)	
38–38.9	79 (23.3)	13 (50.0)	
≥39	15 (4.4)	5 (19.2)	
Cough	179 (52.8)	15 (57.7)	0.63
Sputum production	48 (14.2)	4 (15.4)	0.775
Nasal congestion	4 (1.2)	2 (7.7)	0.061
Fatigue	67 (19.8)	10 (38.5)	0.024
Headache	16 (4.7)	1 (3.8)	>0.999
Sore throat	35 (10.3)	2 (7.7)	>0.999
Shortness of breath	25 (7.4)	3 (11.5)	0.437
Dyspnea	12 (3.5)	1 (3.8)	>0.999
Anorexia	22 (6.5)	5 (19.2)	0.033
Diarrhea	13 (3.8)	2 (7.7)	0.29
Nausea	12 (3.5)	1 (3.8)	>0.999
Myalgia or arthralgia	1 (0.3)	0 (0)	>0.999
Combination of bacterial infection	205 (60.5)	23 (88.5)	0.005
**Treatment**			
Antiviral therapy	326 (96.2)	26 (100.0)	0.611
Antibiotic therapy	228 (67.3)	23 (88.5)	0.025
Use of corticosteroid	109 (32.2)	22 (84.6)	<0.001
Oxygen support	125 (36.9)	24 (92.3)	<0.001
**Clinical outcomes**			
Discharge from hospital	338 (99.7)	25 (96.2)	0.138
Length of hospital stay	14.0 (9.0–20.0)	21.0 (16.0–27.3)	<0.001
Death	1 (0.3)	1 (3.8)	0.138

*Continuous variable data are presented as the mean (standard deviation) or median (interquartile ranges, IQR). Classified variable data are presented as n (%). COPD, Chronic obstructive pulmonary disease*.

To clarify the risk factors for disease progression, univariate and multivariate logistic analyses were conducted (Table 5). Univariate logistic regression identified 13 potential risk factors for disease deterioration. Next, these clinical factors were analysed by multivariate logistic regression, and three variables were retained, suggesting that bilateral patchy shadowing (OR = 4.82, 95% CI: 1.33–17.50; *P* = 0.017) and elevated levels of creatinine (OR = 6.24, 95% CI: 1.42–27.40; *P* = 0.015), and CRP (OR = 7.28, 95% CI: 2.56–20.74; *P* < 0.001) were independent predictors of disease progression. Creatine kinase was statistically significant in the univariate analyses but insignificant in the multivariate analyses. Moreover, ROC curves were used to evaluate the discrimination and predictive ability of creatinine kinase, creatinine, C-reactive protein and bilateral patchy shadowing (Supplementary Figure 1). Results showed that bilateral patchy shadowing, creatinine and CRP obtained AUCs of 0.681, 0.766, 0.806, respectively. Sensitivity and specificity of these parameters at the clinical threshold were also presented. According to ROC analyses, creatinine, C-reactive protein and bilateral patchy shadowing performed well to categorise the patients into severe and non-severe groups.

**Table 5:** Risk factors associated with disease progression among patients with non-severe COVID-19.

	**Univariate**			**Multivariate**		
**Characteristic**	**OR**	**95%CI**	***P*-value**	**OR**	**95%CI**	***P*-value**
Age (≥ 60 years)	3.213	1.408–7.329	0.006			
Gender	0.318	0.130–0.776	0.012			
Hypertension	2.782	1.044–7.415	0.041			
Fatigue	2.537	1.102–5.843	0.029			
Anorexia	3.431	1.181–9.969	0.024			
Bilateral patchy shadowing	7.017	2.068–23.812	0.002	4.822	1.329–17.503	0.017
Lymphocyte count (<0.8 × 10^9^/L)	4.677	1.779–12.291	0.002			
Neutrophil count (>6.4 × 10^9^/L)	4.627	1.776–12.055	0.002			
Platelet count (<100 × 10^9^/L)	0.353	0.145–0.859	0.022			
Creatinine (≥133μmol/L)	10.091	2.651–38.410	0.001	6.241	1.422–27.399	0.015
Lactate dehydrogenase (>245 U/L)	3.904	1.734–8.788	0.001			
Creatinine kinase (>200 U/L)	4.543	1.649–12.517	0.003			
C-reactive protein (≥10 mg/L)	9.363	3.644–24.062	<0.001	7.284	2.558–20.740	<0.001

*OR, odds ratio; CI, confidence interval*.

## Discussion

The majority of patients were diagnosed with mild or moderate COVID-19 upon initial hospitalization. Although they had mild symptoms on admission, they were still at a risk of illness deterioration. There is a medical need to clarify the differences between patients with progressive and non-progressive disease in order to predict which patients are at risk of exacerbation and to conduct early interventions to reduce mortality. In this study, we assessed 365 patients with mild or moderate confirmed COVID-19 on admission. Twenty-six (7.1%) patients progressed to severe or critical disease after admission and were assigned to the progressive disease group, while the others were assigned to the non-progressive disease group. We elucidated the discrepancies in the clinical characteristics between patients with progressive and non-progressive disease and investigated the independent risk factors that could influence disease deterioration, with the purpose to enhance the early identification of at-risk populations.

Many studies have investigated the risk factors associated with disease severity by comparing patients with non-severe COVID-19 and those with severe COVID-19. Liu et al. (10) reported that age, fever, SpO_2_, and cough were linked to severe or critical infections in patients with COVID-19. Zhou et al. (14) proposed that lymphopenia and increased CRP levels were independent risk factors for disease severity. Gong et al. (12) revealed that age, increased CRP, serum lactate dehydrogenase, the coefficient of variation of red blood cell distribution width, albumin, blood urea nitrogen, and direct bilirubin were related to severe COVID-19 and established a predictive tool to distinguish individuals with severe disease. However, in our study, we compared patients with progressive and non-progressive disease, all of whom had mild or moderate COVID-19 on admission. Our study may provide a new perspective on the initial characteristics of patients who will develop severe COVID-19.

Bilateral patchy shadowing and increased levels of creatinine, and CRP were found to be independent predictors of disease progression in this study. Bilateral patchy shadowing is a typical abnormality on chest computed tomography in patients with COVID-19 and has been frequently reported in the literature (15–17). A systematic review of imaging findings in 919 patients with COVID-19 revealed that bilateral involvement was one of the most commonly reported CT findings in severely ill patients (18). Another meta-analysis of radiography findings in patients with SARS, the pathogen of which shares 79.5% of its sequence homology with SARS-CoV-2 (19), found that bilateral involvement of the lungs was the main radiological finding in cases of mortality. These studies verified the role of bilateral patchy shadowing in predicting disease progression to some extent. Elevated creatinine levels were reported to closely correlate with severe COVID-19 (17) or, even worse, with death (1). In another study that attempted to clarify the association between kidney disease and in-hospital death of patients with COVID-19, increased creatinine levels, suggesting kidney injury, were found to represent a higher risk of disease progression in patients with COVID-19 and a greater likelihood of being admitted to the intensive care unit and undergoing mechanical ventilation (20). CRP functions as a non-specific inflammatory marker and is linked to the state of infection and inflammation in patients with severe COVID-19 (21). Prior to CT findings, CRP increased at the early stage of severe COVID-19. CRP level was reported to be associated with CT scores and disease deterioration of COVID-19 (22). The predictive role of CRP for severe COVID-19 has been previously reported (22–24). Increased creatine kinase levels obtained statistical significance in the univariate analyses but lost its position in the multivariate analyses, suggesting that increased creatine kinase levels may be not an independent predictor for disease progression. They are often accompanied by elevated levels of creatine kinase-MB (CK-MB), one of the three forms of creatine kinase and serving as a specific indicator of myocardial injury. The underlying mechanism of how SARS-CoV-2 causes acute myocardial injury is speculated to be related to angiotensin-converting enzyme 2 (25). Interestingly, in a report involving 138 patients hospitalised with COVID-19 in Wuhan, 36 patients who presented with severe manifestations and were treated in the intensive care unit had significantly higher levels of CK-MB than those with non-severe symptoms (1), suggesting that severely ill patients were likely to suffer from acute myocardial injury.

Several limitations existed in the present study. Only 26 patients were included in the group with progressive disease. The relatively small sample size may have had some impact on the results of our study. However, the total population from which these patients were sampled was large at 690. In addition, some clinical indicators such as cytokines were not included because of data unavailability. The role of these indicators may have been underestimated. Moreover, the retrospective nature of the data collection and the risk of potential attribution bias, since the reviewers were not blinded to the final clinical status when they attributed the initial clinical status, remain intrinsic limitations of this study. However, when considering that it is extremely difficult to undertake a prospective collection of data in the context of a COVID-19 outbreak, which still poses a threat to the global community, this method seems appropriate. Additionally, since some previous studies exist in this field, the novelty of this report is limited. Despite these limitations, we still hope our study can contribute to improving the clinical management of disease and benefit patients with COVID-19, especially since so much remains unknown regarding the progression of this disease. More clinical data and further work are urgently needed to define the relevant thresholds and predictive values for the three main variables. Development of predictive tools based on these results to stratify initially hospitalised patients into groups with non-progressive and progressive disease holds great promise and calls for further comprehensive research work.

In conclusion, the patients with progressive disease were significantly different from those with non-progressive disease in terms of demographic features, clinical manifestation, laboratory findings, imaging reports, and clinical outcomes. Bilateral patchy shadowing and elevated levels of creatinine and CRP were found to be independent predictors of disease progression. We hope our study can help elucidate the pathogenic characteristics of patients with COVID-19 and help develop effective biomarkers and further therapeutic strategies for patients with COVID-19.

## Data Availability Statement

The raw data supporting the conclusions of this article will be made available by the authors, without undue reservation.

## Ethics Statement

The studies involving human participants were reviewed and approved by the Medical Ethics Committee of Nanfang Hospital of Southern Medical University and the institutional ethics review boards of all participating hospitals. The requirement for informed consent was waived by the ethics committees.

## Author Contributions

YZ and L-sX designed the research study, analysed the data, and wrote the paper. YZ, L-sX, HongbZ, and PL contributed with literature search and tables. CH and W-FZ contributed with data analysis and writing of the paper. Q-cS, M-yS, S-sL, and W-lZ contributed with data collection. H-yZ and MG performed statistical analyses. LL, Y-LH, and HongZ designed the research study, analysed clinical data, and wrote the paper. All authors contributed to the article and approved the submitted version.

## Conflict of Interest

MG was employed by the Digital China Health Technologies Corporation Limited (Beijing, China). The remaining authors declare that the research was conducted in the absence of any commercial or financial relationships that could be construed as a potential conflict of interest.

## Supplementary Material

Supplementary Figure 1 ROC curves for assessing the predictive value of creatinine kinase, creatinine, C-reactive protein and bilateral patchy shadowing for disease progression. ROC, receiver operating characteristic curve; AUC, area under the ROC curve.
