# Supplementary figures and images for: Clinical Characteristics of Patients With Progressive and Non-progressive Coronavirus Disease 2019: Evidence From 365 Hospitalised Patients in Honghu and Nanchang, China
Source: Front Med (Lausanne). 2020 Nov 16;7:556818. doi: 10.3389/fmed.2020.556818 (PMC7701171; doi:10.3389/fmed.2020.556818)

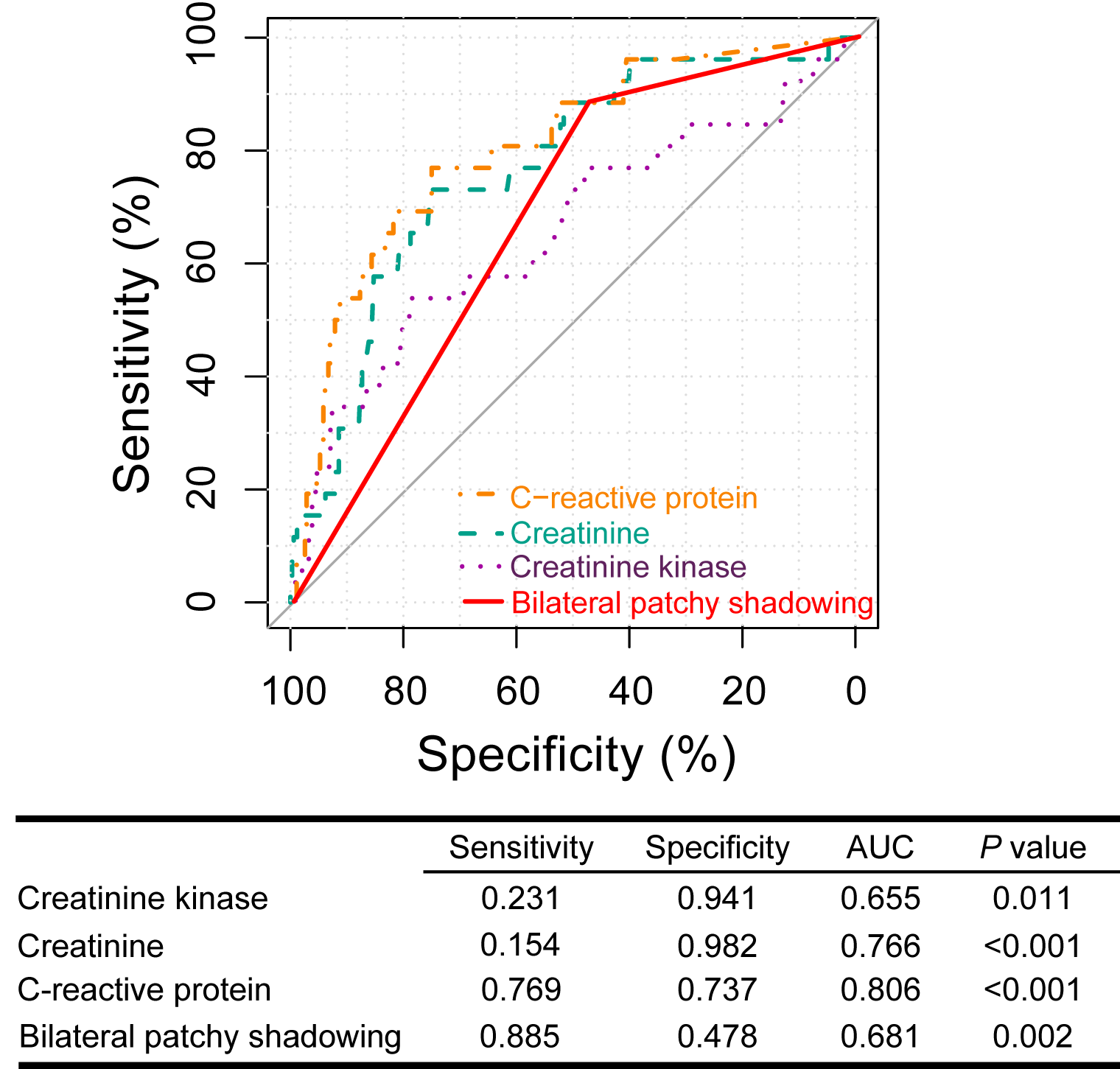

Supplement: Supplementary Figure 1 — ROC curves for assessing the predictive value of creatinine kinase, creatinine, C-reactive protein and bilateral patchy shadowing for disease progression. ROC, receiver operating characteristic curve; AUC, area under the ROC curve. [file Figure_1.TIF]
